# Comparative Biomechanical Analysis of Unilateral, Bilateral, and Lateral Pedicle Screw Implantation in Oblique Lumbar Interbody Fusion: A Finite Element Study

**DOI:** 10.3390/bioengineering10111238

**Published:** 2023-10-24

**Authors:** Chien-Chou Pan, Cheng-Hung Lee, Kun-Hui Chen, Yu-Chun Yen, Kuo-Chih Su

**Affiliations:** 1 Department of Orthopedics, Taichung Veterans General Hospital, Taichung 407, Taiwan; adonisvgh@gmail.com (C.-C.P.); 298f@vghtc.gov.tw (C.-H.L.); orthochen@gmail.com (K.-H.C.); 2 Department of Rehabilitation Science, Jenteh Junior College of Medicine, Nursing and Management, Miaoli 356, Taiwan; 3 Department of Post-Baccalaureate Medicine, College of Medicine, National Chung Hsing University, Taichung 402, Taiwan; 4 Department of Medical Research, Taichung Veterans General Hospital, Taichung 407, Taiwan; yuchunyen@vghtc.gov.tw; 5 Department of Biomedical Engineering, HungKuang University, Taichung 433, Taiwan; 6 Department of Chemical and Materials Engineering, Tunghai University, Taichung 407, Taiwan

**Keywords:** OLIF, finite element analysis, bilateral pedicle screw fixation, unilateral pedicle screw fixation, lateral pedicle screw fixation, biomechanics

## Abstract

Oblique lumbar interbody fusion (OLIF) can be combined with different screw instrumentations. The standard screw instrumentation is bilateral pedicle screw fixation (BPSF). However, the operation is time consuming because a lateral recumbent position must be adopted for OLIF during surgery before a prone position is adopted for BPSF. This study aimed to employ a finite element analysis to investigate the biomechanical effects of OLIF combined with BPSF, unilateral pedicle screw fixation (UPSF), or lateral pedicle screw fixation (LPSF). In this study, three lumbar vertebra finite element models for OLIF surgery with three different fixation methods were developed. The finite element models were assigned six loading conditions (flexion, extension, right lateral bending, left lateral bending, right axial rotation, and left axial rotation), and the total deformation and von Mises stress distribution of the finite element models were observed. The study results showed unremarkable differences in total deformation among different groups (the maximum difference range is approximately 0.6248% to 1.3227%), and that flexion has larger total deformation (5.3604 mm to 5.4011 mm). The groups exhibited different endplate stress because of different movements, but these differences were not large (the maximum difference range between each group is approximately 0.455% to 5.0102%). Using UPSF fixation may lead to higher cage stress (411.08 MPa); however, the stress produced on the endplate was comparable to that in the other two groups. Therefore, the length of surgery can be shortened when unilateral back screws are used for UPSF. In addition, the total deformation and endplate stress of UPSF did not differ much from that of BPSF. Hence, combining OLIF with UPSF can save time and enhance stability, which is comparable to a standard BPSF surgery; thus, this method can be considered by spine surgeons.

## 1. Introduction

Lumbar interbody fusion, including anterior lumbar interbody fusion (ALIF), posterior lumbar interbody fusion (PLIF), transforaminal lumbar interbody fusion (TLIF), lateral lumbar interbody fusion (LLIF), and oblique lumbar interbody fusion (OLIF), is widely used for the treatment of vertebral pseudarthrosis, spinal stenosis or foraminal stenosis, spondylolisthesis, and lateral listhesis [1,2,3,4]. OLIF is a type of minimal invasive surgery (MIS) approach that has the advantages of shorter hospital stays, earlier return to work, decreased intraoperative blood loss, and decreased postoperative pain [1,5,6]. OLIF has superior fusion rates over TLIF, owing to the implantation of cages with a larger footprint [7,8].

However, the standard procedure of OLIF includes implantation of the cage through a retroperitoneal approach in the lateral position and subsequent implantation of pedicle screws through the posterior approach in the prone position. The disadvantages of this procedure include the extra operation room (OR) time and manpower required for reposition, and increased cost of anesthesia, sterile draping, and tools [9]. To overcome these disadvantages, some alternative methods have been described.

Mills et al. performed LLIF combined with posterior pedicle screw insertion in the single-lateral position [10]. This approach has the advantages of decreasing surgery time by approximately 30 min, having a similar screw accuracy as other published articles, and presenting no complications related specifically to single-position surgery. DenHaese et al. performed LLIF with lateral modular plate fixation [11]. The advantages of this approach include single incision (abdomen), which is necessary and saves surgery time. A biomechanical study evaluated the postoperative range of motion (ROM) of the lumbar spine. The authors concluded that compared to LLIF with bilateral pedicle screw implantation, LLIF with lateral modular plate fixation had similar postoperative ROM of axial rotation and lateral bending, but significantly larger ROM of flexion and extension. This implies that LLIF with lateral modular plate fixation might have less flexion–extension stability than LLIF with bilateral pedicle screw fixation (BPSF).

Some studies compared TLIF with either bilateral or unilateral pedicle screw fixation (UPSF). Compared with TLIF with bilateral screw fixation, TLIF with unilateral screw fixation achieved similar clinical outcomes [12,13] and fusion rate [13], fewer surgical injuries and lower cost [12], reduced total blood loss and operation time [13], a significantly lower risk of adjacent segment disease (ASD) [14], and a higher risk of cage migration [13].

Finite element analyses are commonly used in orthopedic biomechanics analyses. Previous studies have employed a finite element analysis to examine the biomechanics of different OLIF fixation methods. Du et al. examined the effects of different degenerative disc diseases (mild, moderate, and severe), using finite element analysis to observe vertebral ROM, intradiscal pressure, facet joint force, stress in the annulus fibrosus and endplate, and other study observation markers [15]. Zhang et al. compared BPSF and UPSF [16]. Although this study observed ROM, it evaluated structural stability after pedicle screw fixation. However, only graft stress was observed when stress on the structure was examined, and that on the vertebra, which spine surgeons were more concerned with, was not observed. Furthermore, this study did not evaluate and compare lateral pedicle screw fixation (LPSF) methods. The results of this study indicated that BPSF provided the best biomechanical stability for OLIF [16]. In addition, Guo et al. also pointed out that BPSF can provide better mechanical stability [17]. However, they only constructed the lumbar vertebrae L3–5 using the computer finite element model, only vertebral implant stress was observed, and bone stress was not examined. Therefore, the condition of the bone after implantation could not be known [17]. Cai et al. showed that BPSF is a better fixation method in OLIF surgeries [18]. Although this study observed endplate values, only L4 or L5 peak stress values were observed, and whether peak stress occurs on the L4 or L5 endplate is unknown. Therefore, further examination of the effects of UPSF on endplate stress in different segments may be required.

The aforementioned studies show that different surgical methods for lumbar OLIF exist. Currently, several special cases in clinical practice require UPSF or LPSF. However, existing studies have not comprehensively examined the effects of endplate stress in different segments, and the mechanical results required by spine surgeons could not be obtained. Therefore, the primary objective of this study was to employ finite element analysis to evaluate the biomechanical effects of three pedicle screw fixation methods (BPSF, UPSF, or LPSF). Concurrently, this study examined the difference in stress in different bone segments under different fixation methods. Thus, this study hypothesized that the stress in bone segments is different between PRSF, UPSF, or LPSF.

## 2. Materials and Methods

### 2.1. Simulation of Lumbar Geometry Model

To evaluate the effects of three different lumbar vertebral OLIF surgical fixation methods, this study used the computed tomography (CT) images of an artificial lumbar vertebrae model for finite element analysis of the computer lumbar vertebrae model. A commercially available artificial lumbar vertebrae model (SKU:1352, Pacific Research Laboratories, Inc., Vashon, Washington, DC, USA) was used to obtain CT images using CT scanning and the medical image reconstruction software Mimics (Mimics Medical 21.0, Materialise, Leuven, Belgium). Grayscale values were used to select bones in the vertebral model in CT images, and a computer lumbar vertebrae model was constructed.

**Figure 1:**
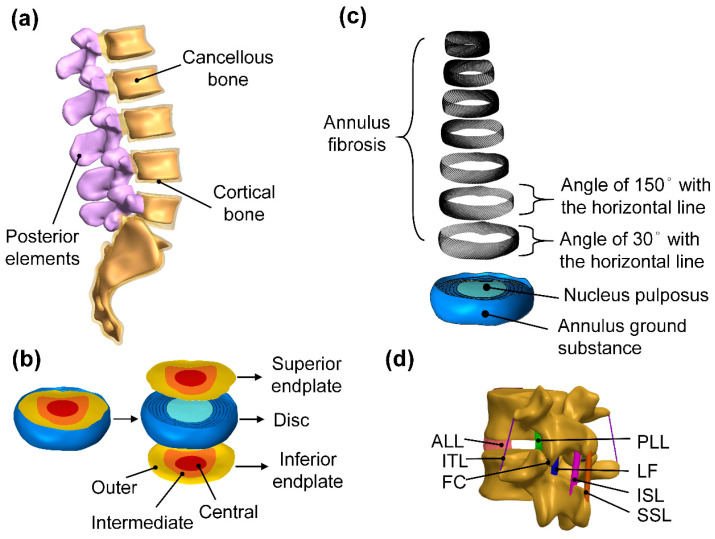
Parts of the lumbar spine computer model. (**a**) Cortical bone, cancellous bone, and posterior elements. (**b**) Disc and endplate details. (**c**) Intervertebral disc (annulus ground substance and annulus fibrosus). (**d**) Ligaments.

Mimics was used to construct a lumbar vertebrae model. Subsequently, Geomagic Design X (Geomagic Design X, 3D Systems, Rock Hill, South Carolina, SC, USA) was used to convert the computer lumbar vertebrae model to an igs file. Then, this igs file was imported into the three-dimensional computer-assisted design software (Solidworks 2016, Dassault Systemes SolidWorks Corp, Waltham, MA, USA) and was drawn and segmented (Figure 1). In this study, the vertebrae model was divided into several parts, including the cortical bone, cancellous bone, endplate (central, intermediate, and outer endplate), posterior elements, annulus ground substance, nucleus pulposus, annulus fibrosus, anterior longitudinal ligament (ALL), posterior longitudinal ligament (PLL), interspinous ligament (ISL), supraspinous ligament (SSL), intertransverse ligament (ITL), ligamentum flavum (LF), and facet capsulary ligament (FC). For the disc, seven layers of annulus ground substance and seven layers of annulus fibrosus were constructed. The intervertebral disc consists of annulus fibrosus and nucleus pulposus, of which the annulus fibrosus was formed through alternating annulus ground substance and circular fibers. Each layer of circular fiber was surrounded by fibers inclined at an angle of 30° or 150° to the horizontal line.

### 2.2. Different Pedicle Screw Systems

**Figure 2:**
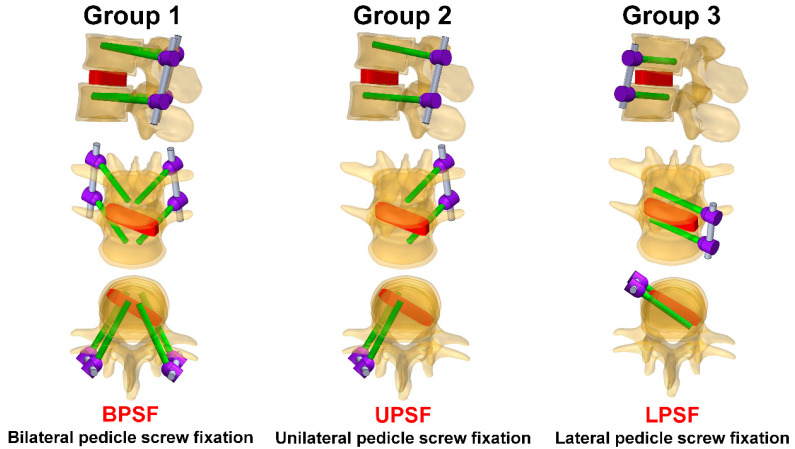
Three groups in this study. The fixation conditions are BPSF, UPSF, and LPSF.

For the pedicle screw, three computer models with different pedicle screw systems implanted in the lumbar vertebrae were constructed. The pedicle screw system mainly comprised the pedicle screw (5.5 mm in diameter and 45 mm in length), screw tulip, and rod. In addition, the cage was constructed and inserted between the L3 and L4 intervertebral discs in the lumbar vertebrae model, which was used to simulate vertebral arch implantation and fixation. This study mainly examined three pedicle screw fixation methods in the lumbar vertebrae L3 and L4. The three fixation method groups were as follows: Group 1 (BPSF): BPSF, in which traditional pedicle screws were implanted in the posterior side of L3 and L4; Group 2 (UPSF): the unilateral pedicle screw fixation, in which unilateral pedicle screws were implanted in the posterior side of L3 and L4; and Group 3 (LPSF): the lateral pedicle screw fixation, in which traditional pedicle screws were implanted in the left anterior side of L3 and L4 (Figure 2).

The three computer models were then imported into the finite element analysis software ANSYS Workbench (ANSYS Workbench 18.0, ANSYS, Inc., Canonsburg, PA, USA) for finite element analysis.

### 2.3. Loading and Boundary Conditions

**Figure 3:**
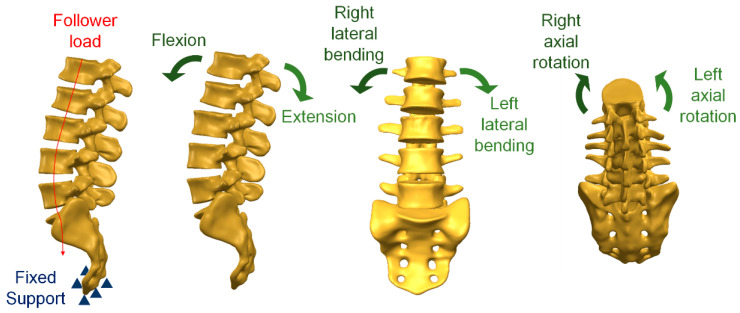
Loading and boundary conditions.

For setting loading and boundary conditions, a previous study [19] was used to perform six types of movements (flexion, extension, left and right lateral bending, and left and right axial rotation) in the lumbar vertebrae. Therefore, for loading conditions, different follower loads and motions were used for flexion, extension, left and right lateral bending, and left and right axial rotation. In the simulated flexion movement, a follower load of 1175 N and motion of 7.5 Nm were set. In the simulated extension movement, a follower load of 500 N and motion of 7.5 Nm were set. In the simulated lateral bending movement, a follower load of 700 N and motion of ±7.8 Nm were set. In the simulated axial rotation movement, a follower load of 720 N and motion of ±5.5 Nm were set. In addition, the boundary conditions were the lower edges (*X*-, *Y*-, and *Z*-axis displacements were used) for the fixed sacral model (Figure 3).

### 2.4. Material Properties of the Model

**Figure 4:**
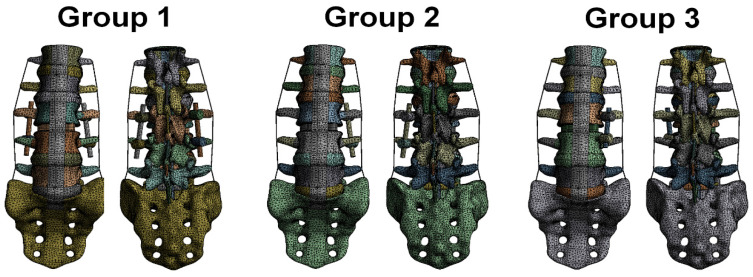
Mesh condition of three finite element analysis models in this study.

Previous studies [20,21,22,23,24,25,26] were used as a reference for setting up lumbar vertebrae model material characteristics in the finite element analysis software. Titanium alloys were used to simulate the cage and pedicle system. The assumptions were that all materials were linearly elastic, homogeneous, and isotropic. Therefore, Young’s modulus and Poisson’s ratio were used to represent the material characteristics of the lumbar vertebrae and implant in the material characteristic setting. Table 1 shows the material properties used in this finite element study. Furthermore, the mesh used in the computer model for finite element analysis was tetrahedral mesh (Figure 4). To achieve better calculation accuracy in the lumbar vertebrae computer model used in this study, the meshing of the lumbar vertebrae model was ensured to pass the mesh convergence test and reach 5% of the stop criterion of the convergence test. Hence, the finite element model used in this study was rational. The mesh used in this study was 2 mm. Table 2 presents the numbers of nodes and elements used in this study.

The observation markers in this study were the total deformation of the lumbar spine, von Mises stress of lumbar spines, and von Mises stress of the pedicle screw system. The biomechanical effects of three different pedicle screw fixation methods were evaluated.

## 3. Results

**Figure 5:**
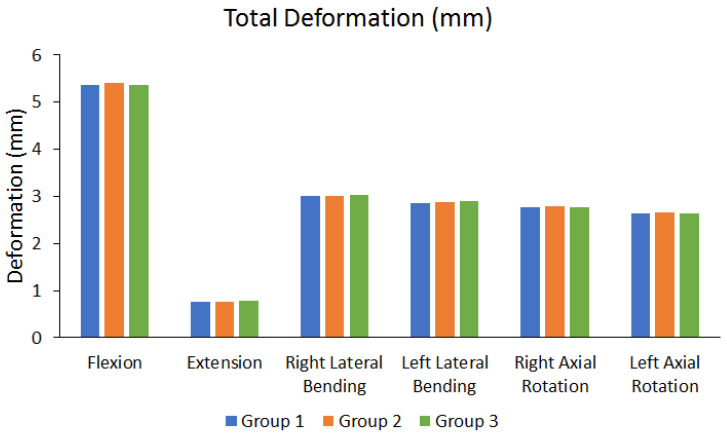
Total deformation under six movements.

This study employed a finite element analysis to examine the biomechanical effects of OLIF with either BPSF, UPSF, or LPSF. Figure 5 mainly shows the overall total deformation results of different groups under six different movements. The total deformation was higher under flexion (5.3604 mm to 5.4011 mm) and lower under extension (0.76303 mm to 0.77297 mm). In addition, the differences among the values of different groups were not large (the maximum difference range is approximately 0.6248% to 1.3227%). Table 3 shows the peak total deformation value of each group, and the percentage of the maximum difference between each group.

**Figure 6:**
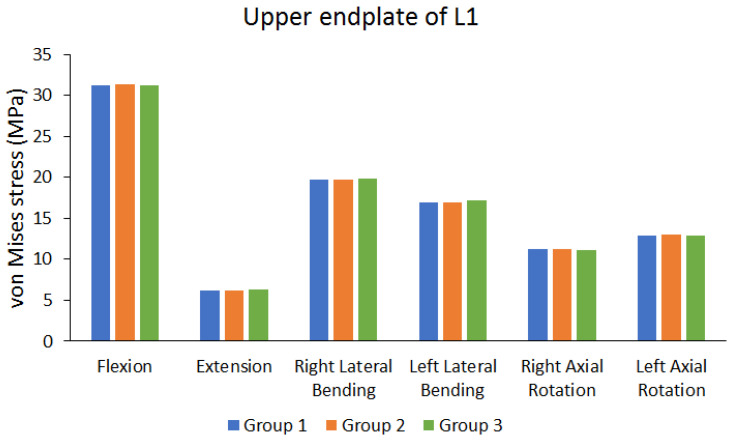
Peak von Mises stress of the upper endplate of the first lumbar (L1) vertebra.

**Figure 7:**
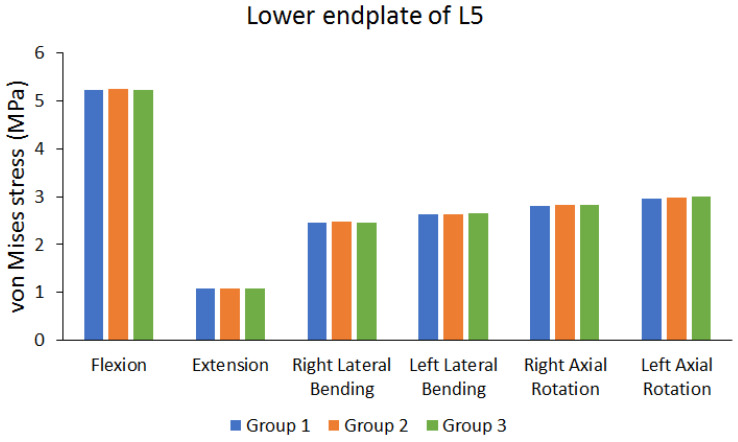
Peak von Mises stress of the lower endplate of the fifth lumbar (L5) vertebra.

Figure 6 shows the von Mises stress results of the upper endplate of the first lumbar (L1) vertebra. Figure 7 shows the von Mises stress results of the lower endplate of the fifth lumbar (L5) vertebra. Different von Mises stresses were observed on the upper endplate of L1 and lower endplate of L5 under different movements in different groups. Flexion resulted in larger von Mises stress, whereas extension resulted in lower von Mises stress. In addition, the differences in von Mises stress of different groups were not large (the maximum difference range is approximately 0.6498% to 2.6186%). Table 4 shows the endplate von Mises stress values of each group, and the percentage of the maximum difference between each group.

**Figure 8:**
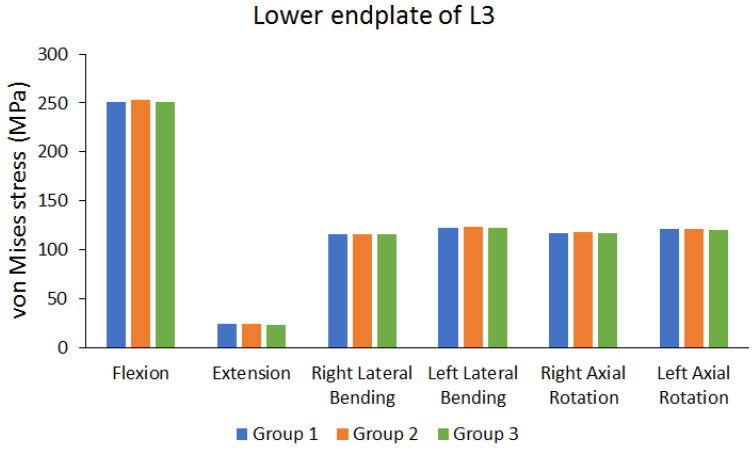
Peak von Mises stress of the lower endplate of the third lumbar (L3) vertebra.

**Figure 9:**
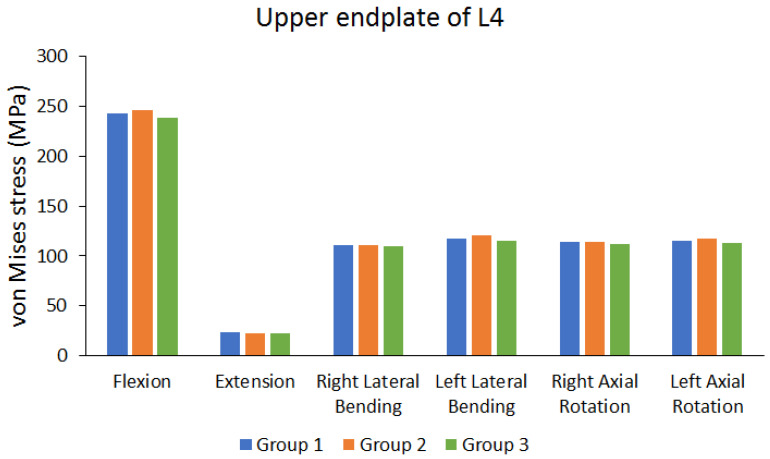
Peak von Mises stress of the upper endplate of the fourth lumbar (L4) vertebra.

The cage was implanted between L3 and L4. Therefore, and the von Mises stress in the endplate near the cage was observed. Figure 8 shows the von Mises stress of the lower endplate of L3. Figure 9 shows the von Mises stress results of the upper endplate of L4. The results show the von Mises stress on the lower endplate of L3 and upper endplate of L4 under different movements in various groups. Flexion resulted in larger von Mises stress, whereas extension resulted in lower von Mises stress. In addition, these values were larger than the stress on other lumbar vertebral segments. The differences in von Mises stress values of different groups were not large (the maximum difference range is approximately 0.9235% to 4.4781%).

**Figure 10:**
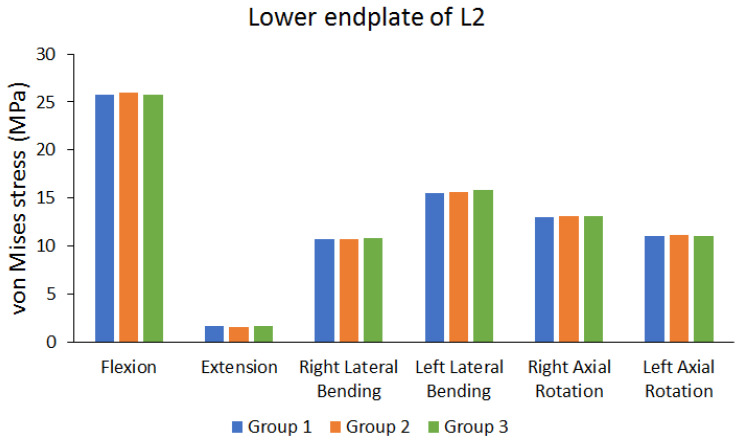
Peak von Mises stress of the lower endplate of the second lumbar (L2) vertebra.

**Figure 11:**
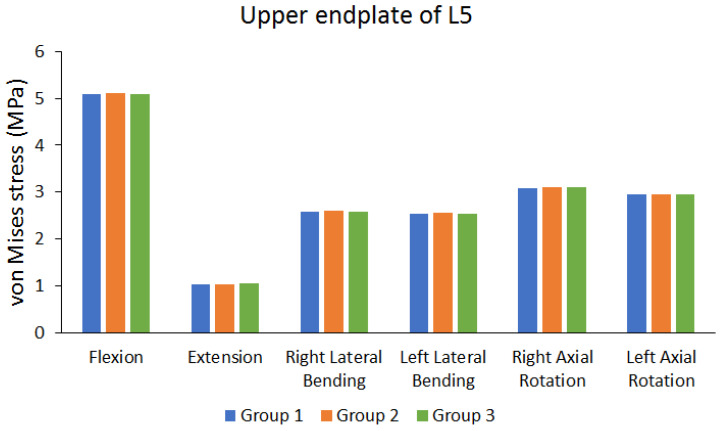
Peak von Mises stress of the upper endplate of the fifth lumbar (L5) vertebra.

The cage was implanted in the disc space between the third and fourth lumbar vertebrae. The von Mises stress in the endplate near the cage was observed. Figure 10 shows the von Mises stress results of the lower endplate of the second lumbar (L2) vertebra. Figure 11 shows the von Mises stress results of the upper endplate of the fifth lumbar (L5) vertebra. The results show the von Mises stress on the lower endplate of L2 and upper endplate of L5 under different movements in different groups. Flexion resulted in larger von Mises stress, whereas extension resulted in lower von Mises stress. The differences in the von Mises stress values of different groups are not large. (the maximum difference range is approximately 0.455% to 5.0102%).

**Figure 12:**
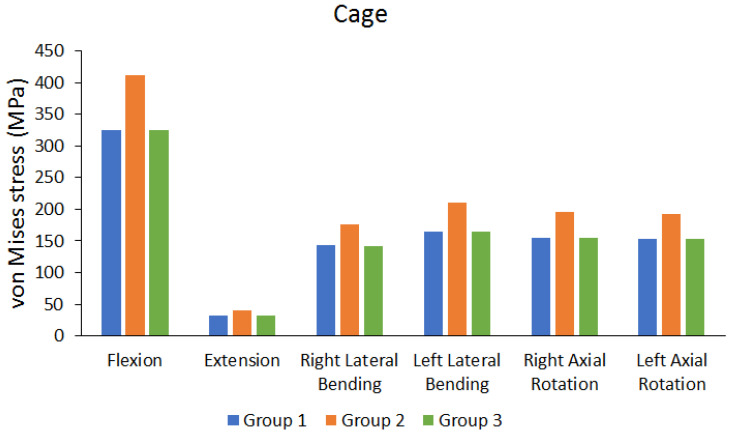
Peak von Mises stress of the cage.

Figure 12 shows the von Mises stress on the cage. The results showed that the cage experienced greater stress when UPSF was used. The differences in von Mises stress were not high when BPSF and LPSF were used (the difference is approximately 0.0518% to 0.3280%). Table 5 shows the peak cage and screws von Mises stress values of each group, and the percentage of the difference.

**Figure 13:**
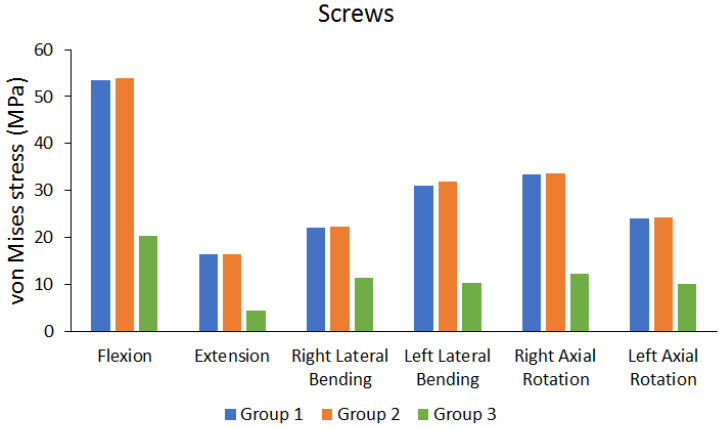
Peak von Mises stress of the screws.

Figure 13 shows the von Mises stress on the screw when BPSF, UPSF, and LPSF were used. The results showed that the differences in von Mises stress on the screw are not high when UPSF and BPSF were used (the difference is approximately 0.3773% to 2.5416%). The stress on the screw was lower when LPSF was used, compared to that when UPSF and BPSF were used.

## 4. Discussion

This study mainly evaluated the biomechanical effects of three different pedicle screw fixation methods (BPSF, UPSF, LPSF) when an OLIF cage was used in lumbar interbody fusion. A finite element analysis was used for the simulation of the vertebral model under three different fixation methods, and to evaluate the total deformation and stress distribution of the different methods. In the procedural workflow of finite element analysis employed in this study, the primary steps included the initial creation of a computer model of the spine. Subsequently, the computer model underwent mesh generation to facilitate segmentation, followed by the specification of material properties to mimic spinal characteristics. Computational numerical analysis techniques were then utilized to solve the model. Finally, a comprehensive biomechanical evaluation of parameters of interest was conducted. Because the structure of the spine is quite complex, finite element analysis was a suitable method for our research to evaluate it thoroughly.

Observation of total deformation of the vertebrae under BPSF, UPSF, or LPSF showed that the stress experienced by the three different fixation methods was different during six movements (flexion, extension, left and right lateral bending, and left and right axial rotation), which is primarily because the differences between the movements were larger, resulting in different deformations. Furthermore, total deformation was observed to be larger under flexion and lower under extension, which is primarily because the extension is affected by restriction in the posterior vertebra (facet joint) and therefore has lower displacement deformation. In addition, the differences among the groups were not large under the same movement, which is primarily because the implantation region in this study was L3–4; therefore, the total deformation difference was not large. According to Hooke’s law, stress = Young’s modulus×strain, and deformation is associated with strain. Therefore, under the same movement, as Young’s modulus of the endplate used in different groups is the same, the maximum difference in the stress between the upper endplate of L1 and lower endplate of L5 was not large.

We observed the stress of the endplate near L3–4 under identical movements, and found that the maximum stress difference between the lower endplate of L3 and upper endplate of L4 was not large, which is primarily because the difference in deformation among the groups was not large under BPSF, UPSF, or LPSF; therefore, the stress difference in endplates near L3–4 was low. Similarly, we observed the maximum stress of endplates near the segments, and found that the maximum stress difference between the lower endplate of L2 and upper endplate of L5 was low. Furthermore, observation of the stress on the implanted cage showed that under the same movements, the UPSF implantation method resulted in greater stress on the cage, which is primarily because two pedicle screws were used for fixation in BPSF, and this produces external protective effects on the case, thereby decreasing cage deformation and resulting in low cage stress. The trend in this study’s results is consistent with other previous studies [17,18]. Moreover, the direction of lateral pedicle screw implantation under LPSF is almost parallel to the cage. Therefore, the protection provided by the lateral pedicle screw decreases cage deformation, thereby resulting in lower cage stress. However, the results of this study are different from previous research trends [17]. The main reason may be that the cage and cage placement used in this study are different from previous studies, resulting in different results. Although the stress on the cage is greater when UPSF is used, this did not affect endplate (L3–4 and neighboring segments) stress, and the stress of this cage was much lower than the yield strength (1100 MPa) [27]. Therefore, using UPSF fixation does not cause the cage to be easily destroyed.

Observation of stress on the screw showed that the stress was lower when LPSF was used for the same movements. Using BPSF or UPSF causes greater stress on the screw; however, these differences are not large. The reason for the lower stress under LPSF is that the screw is parallel to the cage. This causes the deformation on the screw to be insignificant, thereby decreasing strain and resulting in lower stress. Furthermore, the stress on the screw is different because the location of lateral pedicle screw fixation when LPSF is used is different from that under BPSF and UPSF. In addition, the stress on the screws under the three different fixation methods was much lower than the yield strength.

OLIF with lateral modular plate fixation is an alternative method to provide similar axial and lateral stability, but a larger flexion–extension ROM [11]. This approach does not need an additional back wound for pedicle screw implantation, and thus has shorter surgical time. However, due to weaker stability of lumbar flexion–extension, this surgery is usually not recommended for patients with spondylolisthesis of grade II or higher. If surgeons need to perform OLIF for patients experiencing spondylolisthesis of grade II or higher, OLIF plus posterior pedicle screw implantation is a better choice. However, this procedure is time-consuming compared with TLIF [9]. Our study revealed that the stress over the bone is similar between the BPSF and UPSF groups. This implies that OLIF plus UPSF might be applied to patients who are indicated for ding OLIF plus BPSF. In addition, OLIF plus UPSF may provide enough vertebral stability, like OLIF plus BPSF, and might decrease surgical time, blood loss, and radiation exposure. In addition, previous research indicates that in order to decrease the risks associated with BPSF surgery, some surgeons selected UPSF, which can provide better stability and reduce surgical costs for patients [16]. Such an argument is similar to the present study.

This study has some limitations. As the lumbar vertebrae model used in this study has a more complex structure, common settings were used for orthopedic materials in finite element analysis. The materials used in this study were set as homogeneous, isotropic, and linearly elastic materials [28,29]. Such material property settings may have slight differences from real conditions, primarily due to the fact that real spines are heterogeneous and anisotropic. Additionally, these material property settings assume the conditions of generally healthy bones, and may not represent cases of osteoporosis or similar conditions. Furthermore, this study’s computer model only includes lumbar vertebrae and the sacrum, and does not incorporate structures such as muscles, skin, soft tissues, and others. This is mainly because the computer model used in this study already involves a significant number of components, and creating a complete spinal computer model might require more time and computational resources. The simplifications made in the material property settings and computer modeling in this study were primarily to avoid altering too many variables, which could impact the primary observed parameters of this research. However, it is important to note that these simplifications do not alter the trends in the study’s results. Despite some differences from real scenarios, the trends in the research findings can still provide biomechanical guidance for spine surgeons in selecting surgical approaches.

This study employed finite element analysis to evaluate the effects of three different pedicle screw implantation methods in OLIF surgery and examined the correlation between OLIF and BPSF, UPSF, and LPSF. However, some potential directions for future studies, such as using unilateral pedicle screws for multi-segment fixation, and lateral pedicle screws for multi-segment fixation, remain. The biomechanics of these different fixation methods are of interest to spine surgeons. However, the vertebra model construction in this study can be used in the future for further finite element analyses and biomechanical evaluation of vertebral fixation. Beyond this finite element study, future studies such as sawbones study, cadaver studies, clinical studies, and biomechanical evaluation of other spinal implant designs are necessary for confirmation of clinical applications and outcomes.

## 5. Conclusions

Though OLIF plus LPSF does not need an additional back wound for pedicle screw implantation and is a time-saving procedure, it could not be applied to patients with spondylolisthesis of grade II or higher due to weaker lumbar flexion–extension stability. Up to now, BPSF is still a mainstream supplementary fixation for OLIF. This study employed finite element analysis to evaluate the biomechanical effects of three different pedicle screw fixation methods (BPSF, UPSF, or LPSF) combined with lumbar vertebral OLIF surgery. The study revealed that the total deformation and endplate stress of UPSF did not differ much from BPSF. For spine surgeons, OLIF plus UPSF provides similar stability of OLIF plus BPSF, diminishes surgical wounds, and might decrease surgical time and intraoperative radiation exposure.

## Figures and Tables

**Table 1:** Material properties used in the finite element analysis of this study [20,21,22,23,24,25,26].

Materials	Young’s Modulus (MPa)	Poisson’s Ratio
Cortical bone	12,000	0.3
Cancellous bone	100	0.3
Endplate: central	2000	0.3
Endplate: intermediate	6000	0.3
Endplate: outer	12,000	0.3
Posterior elements	3500	0.25
Nucleus pulposus	1	0.499
Annulus fibrosus 1–2 (outermost layers)	550	0.3
Annulus fibrosus 3–4	485	0.3
Annulus fibrosus 5–6	420	0.3
Annulus fibrosus 7 (innermost layer)	360	0.3
Annulus ground substance	4.2	0.45
Anterior longitudinal ligament	20	0.3
Posterior longitudinal ligament	20	0.3
Ligamentum flavum	19.5	0.3
Interspinous ligament	11.6	0.3
Supraspinous ligament	15	0.3
Intertransverse ligament	58.7	0.3
Facet capsulary ligament	32.9	0.3
Titanium alloy	110,000	0.3

**Table 2:** The number of nodes and the number of elements used in this study.

Mesh	Group 1	Group 2	Group 3
Number of nodes	1,625,598	1,611,840	1,611,185
Number of elements	395,903	389,745	389,648

**Table 3:** Peak total deformation value of each group and the percentage of the maximum difference between each group.

		Flexion	Extension	Right Lateral Bending	Left Lateral Bending	Right Axial Rotation	Left Axial Rotation
Total Deformation	Group 1 (mm)	5.3623	0.76322	3.0041	2.8574	2.768	2.6459
	Group 2 (mm)	5.4011	0.76303	3.0198	2.8786	2.785	2.6634
	Group 3 (mm)	5.3604	0.77297	3.0291	2.8957	2.7676	2.6454
	The maximum difference between each group (%)	0.7536%	1.2859%	0.8253%	1.3227%	0.6248%	0.6758%

**Table 4:** Peak endplate von Mises stress values of each group and the percentage of the maximum difference between each group.

		Flexion	Extension	Right Lateral Bending	Left Lateral Bending	Right Axial Rotation	Left Axial Rotation
Peak von Mises stress of upper endplate of L1	Group 1 (MPa)	31.193	6.2037	19.694	16.878	11.184	12.9
	Group 2 (MPa)	31.394	6.1918	19.764	16.978	11.258	12.978
	Group 3 (MPa)	31.19	6.3583	19.843	17.135	11.137	12.889
	The maximum difference between each group (%)	0.6498%	2.6186%	0.7509%	1.4999%	1.0748%	0.6858%
Peak von Mises stress of lower endplate of L5	Group 1 (MPa)	5.2205	1.0727	2.4574	2.6281	2.8074	2.9604
	Group 2 (MPa)	5.257	1.0757	2.4719	2.6401	2.8263	2.9819
	Group 3 (MPa)	5.2374	1.0798	2.4524	2.6613	2.835	2.9952
	The maximum difference between each group (%)	0.6943%	0.6575%	0.7889%	1.2475%	0.9735%	1.1619%
Peak von Mises stress of lower endplate of L3	Group 1 (MPa)	251.31	23.539	115.35	122.35	116.83	120.65
	Group 2 (MPa)	253.42	23.513	116.02	123.44	117.71	121.68
	Group 3 (MPa)	250.76	23.262	115.47	122.3	116.47	120.21
	The maximum difference between each group (%)	1.0496%	1.1768%	0.5775%	0.9235%	1.0534%	1.2081%
Peak von Mises stress of upper endplate of L4	Group 1 (MPa)	242.97	23.481	111.22	117.77	114.51	115.01
	Group 2 (MPa)	245.92	22.697	110.92	120.81	114.1	117.76
	Group 3 (MPa)	238.01	22.884	109.48	115.4	112.2	112.54
	The maximum difference between each group (%)	3.2165%	3.3389%	1.5645%	4.4781%	2.0173%	4.4327%
Peak von Mises stress of lower endplate of L2	Group 1 (MPa)	25.755	1.6286	10.657	15.469	13.041	11.019
	Group 2 (MPa)	25.972	1.5774	10.718	15.634	13.119	11.097
	Group 3 (MPa)	25.801	1.6606	10.781	15.818	13.099	11.01
	The maximum difference between each group (%)	0.8355%	5.0102%	1.1502%	2.2063%	0.5946%	0.7840%
Peak von Mises stress of upper endplate of L5	Group 1 (MPa)	5.0849	1.0358	2.5806	2.5379	3.0743	2.9458
	Group 2 (MPa)	5.1124	1.0392	2.5949	2.5495	3.0928	2.9612
	Group 3 (MPa)	5.0833	1.0416	2.5756	2.5466	3.0932	2.9483
	The maximum difference between each group (%)	0.5692%	0.5568%	0.7438%	0.4550%	0.6110%	0.5201%

**Table 5:** Peak cage and screws’ von Mises stress values of each group, and the percentage difference.

		Flexion	Extension	Right Lateral Bending	Left Lateral Bending	Right Axial Rotation	Left Axial Rotation
Peak von Mises stress of cage	Group 1 (MPa)	325.74	31.45	142.67	164.1	154.33	152.95
	Group 2 (MPa)	411.08	39.45	176.14	210.17	194.98	192.62
	Group 3 (MPa)	325.34	31.419	142.37	164.64	154.25	152.66
	Differences between BPSF and LPSF Group (%)	0.1228%	0.0986%	0.2103%	0.3280%	0.0518%	0.1896%
Peak von Mises stress of screws	Group 1 (MPa)	53.47	16.433	21.978	30.983	33.401	24.073
	Group 2 (MPa)	53.857	16.371	22.307	31.791	33.692	24.272
	Group 3 (MPa)	20.376	4.4699	11.28	10.22	12.327	10.127
	Differences between UPSF and BPSF Group (%)	0.7186%	0.3773%	1.4749%	2.5416%	0.8637%	0.8199%

## Data Availability

Not applicable.
